# C1-C2 fractures in asymptomatic elderly patients with minor head trauma: evaluation with a dedicated head CT protocol

**DOI:** 10.1590/0100-3984.2017.0154

**Published:** 2019

**Authors:** Silvia Squarza, Carla Luisa Uggetti, Marco Angelo Politi, Lorenzo Carlo Pescatori, Raffaele Bisogno, Adriana Campi, Paolo Reganati, Maurizio Cariati

**Affiliations:** 1 Neuroradiology Unit, Radiology Department, Azienda Socio Sanitaria Territoriale Santi Paolo e Carlo, San Carlo Borromeo Hospital, Milano, Italy.; 2 Neuroradiology Unit, Cannizzaro Hospital, Catania, Italy.; 3 Graduate School of Radiodiagnostics, University of Milan, Milano, Italy.; 4 San Carlo Clinic, Paderno Dugnano, Italy.

**Keywords:** Craniocerebral trauma, Aged, Aged, 80 and over, Spinal injuries/diagnosis, Cervical vertebrae/injuries, Tomography, X-ray computed., Traumatismos craniocerebrais, Idoso, Idoso de 80 anos ou mais, Traumatismos da coluna vertebral/diagnóstico, Vértebras cervicais/lesões, Tomografia computadorizada

## Abstract

**Objective:**

To evaluate the frequency and types of upper cervical spine injuries in asymptomatic elderly patients undergoing computed tomography (CT) for the investigation of minor head trauma.

**Materials and Methods:**

This was a prospective study of 2613 asymptomatic elderly patients with minor head trauma seen between January 2015 and December 2016. We adopted a dedicated head CT protocol that included the C1-C2 region.

**Results:**

Of the 2613 patients analyzed, 33 (1.26%) had upper cervical spine injuries, corresponding to 8.37% of the 394 patients with trauma-related findings. Of those 33 patients, 6 had C1 fractures and 27 had C2 fractures. The use of 16- and 128-slice scanners increased the CT dose by 25.0% and 23.7%, respectively.

**Conclusion:**

Inclusion of the C1-C2 region in head CT scans allowed us to identify upper cervical spine injuries in 1.26% of asymptomatic elderly patients with minor head trauma. The protocol evaluated helps detect potentially life-threatening injuries and could be adopted for routine use in elderly individuals with minor head trauma.

## Abstract (pt)

**Objetivo:**

Avaliar a frequência e os tipos de lesões da coluna cervical superior em pacientes idosos assintomáticos submetidos a tomografia computadorizada (TC) para investigação de trauma leve na cabeça.

**Materiais e Métodos:**

De janeiro de 2015 a dezembro de 2016, analisamos prospectivamente 2613 pacientes idosos assintomáticos com pequeno traumatismo na cabeça. com protocolo de TC dedicado incluindo a região de C1-C2.

**Resultados:**

Trinta e três dos 2613 pacientes apresentaram lesões na coluna cervical superior, com frequência de 1,26% em toda a população e de 8,37% (33/394) em pacientes com achados relacionados ao trauma. Seis dos 33 pacientes apresentaram fratura de C1 e 27/33 pacientes apresentaram fratura de C2. A dose de TC aumentou 25% e 23,68% com *scanner* de 16 e 128 fileiras, respectivamente.

**Conclusão:**

A inclusão de C1-C2 na TC de cabeça revelou uma taxa de lesões da coluna cervical superior de 1,26% em pacientes idosos assintomáticos com lesão pequena na cabeça. O protocolo ajuda a detectar potencialmente lesões fatais e pode ser adotado para pessoas idosas com trauma leve na cabeça.

## INTRODUCTION

Traumatic brain injury is a major cause of death and disability, in young people and in the elderly. The increase in active life expectancy has created a surge in the number of elderly trauma patients-elderly being defined as ≥ 65 years of age-especially those with minor head trauma. The severity of head trauma is evaluated with the Glasgow Coma Scale (GCS), on which a score ≥ 13 is indicative of minimal or no alterations in mental status and consequently of minor head trauma^(^1^,^^2^^)^.

For adult patients with minor head trauma presenting to the emergency department, there is no consensus regarding the use of computed tomography (CT) of the head, although several guidelines are available. Elderly patients merit special consideration because they are considered to be at major risk for intracranial complications and there is more agreement about the indications to perform head CT^(^3^)^.

In the literature, there is considerable evidence that the frequency of upper cervical spine trauma, especially atlantoaxial fractures, is high among the elderly, due to reduced bone density, different mechanisms of injury, and degenerative changes affecting the biomechanics of the spine. In fact, degenerative changes cause stiffening of the cervical spine leading to decreased mobility and consequently to a higher incidence of upper cervical spine injuries^(^4^,^^5^^)^.

Mortality rates as high as 25-30% have been reported after odontoid fractures in elderly patients^(^6^)^. In such patients, symptoms can be mild or absent. Cervical X-ray can be difficult to perform or poorly diagnostic in comparison with CT, which is considered the most cost-effective technique^(^7^,^^8^^)^.

The most important international guidelines for minor head trauma are the New Orleans criteria^(^9^)^, published in 2000; the Canadian rule^(^10^,^^11^^)^, published in 2001; and the National Institute for Health and Clinical Excellence (NICE) guidelines^(^12^)^, issued in 2014 and revised in 2017. According to the NICE guidelines in particular, CT of the head is considered the examination of choice to detect acute clinically important brain injuries. CT is recommended specifically for adults with any of the following risk factors: a GCS score < 13 in the initial assessment or < 15 at 2 h after admission; suspected open or depressed skull fracture or sign of fracture of the skull base; seizure; focal neurological deficit; and more than one episode of vomiting. Head CT should also be performed in adults who have experienced some loss of consciousness or amnesia and present any of the following risk factors: age ≥ 65 years; any history of bleeding or clotting disorder; and a dangerous mechanism of trauma.

Considering the investigation of cervical spine injuries, the NICE guidelines specifically state the following^(^12^)^: "In CT, routinely review on 'bone windows' the occipital condyle region for patients who have sustained a head injury. Reconstruction of standard head images onto a high-resolution bony algorithm is readily achieved with modern CT scanners." The various guidelines also state that cervical spine CT is recommended if patients are ≥ 65 years of age and symptomatic^(^9^-^12^)^.

To our knowledge, there have been few reports regarding the incidence of fracture of the upper cervical spine (C1-C2) in asymptomatic elderly individuals presenting to the emergency department with minor head trauma. We decided to investigate the frequency of unexpected upper cervical spine injuries in elderly patients with minor head trauma who underwent head CT. With that aim, we included the first two cervical vertebrae in head CT scans performed in elderly patients with minor head trauma. In addition, we compared patients who were 65-75 years of age with those who were over 75 years of age, in terms of the frequency of upper cervical spine injury, in order to investigate a possible age-related difference. Finally, we analyzed the effective dose in our proposed head CT protocol in comparison with that of a standard head CT protocol.

## MATERIALS AND METHODS

### Patients

Over a period of 36 months (between January 2015 and December 2016), we prospectively analyzed all head CT scans performed in our emergency department in patients ≥ 65 years of age with minor head trauma. This type of imaging procedure was approved by the research ethics committee of our institution.

Patients who were symptomatic for upper cervical spine injuries were excluded. To define the presence or absence of symptoms, we considered the first evaluation (i.e., that performed at admission to our emergency department). Patients who did not report neck pain were categorized as asymptomatic. Because those patients presented with minor head trauma without cervical pain, a specific evaluation of cervical motility was not routinely performed. A total of 2613 patients admitted to our emergency department for minor head trauma underwent a specific head CT protocol with inclusion of the upper cervical spine, the first two cervical vertebrae in particular. We employed two different multidetector scanners: a 16-slice scanner (Lightspeed 16; GE Medical Systems, Milwaukee, WI, USA) and a 128-slice scanner (Somatom Definition AS; Siemens Medical Systems, Forchheim, Germany). Thin-slice reconstructions with a bone algorithm and multiplanar reconstructions were always obtained to identify post-traumatic bone lesions.

### Data analysis

Images and clinical data of patients with upper cervical spine injuries were analyzed by four neuroradiologists (with 10-30 years experience). Four different radiologists (each with at least 4 years experience) read the CT reports, reporting all data that were relevant for the diagnosis and related to the trauma. General characteristics and CT findings were evaluated for each patient. The association of upper cervical spine injuries with the type and site of trauma, as well as with the therapeutic approach used (conservative or surgical), was examined. We evaluated the frequency of post-traumatic injuries within the overall population, among patients 65-75 years of age, and among patients over 75 of age.

### Classification of C1-C2 fractures

In the evaluation of the CT images, we classified the upper cervical spine injuries as C1 or C2 fractures. Fractures of the C1 vertebra include (vertical or transverse) fracture of the anterior arch, bilateral fractures of the anterior arch with posterior atlantoaxial dislocation, fracture of the lateral mass, fracture of the posterior arch, and Jefferson fracture. A Jefferson fracture is a burst fracture of C1, described as a two-, three-, or four-part fracture involving the anterior and posterior arches. CT of such fractures typically demonstrates a fracture line involving the anterior and posterior arches, whereas the transverse ligament is often intact^(^13^)^. The typical C2 fracture is odontoid fracture, which is defined as a fracture involving the odontoid process of C2. The most common odontoid fracture classification, the Anderson and D'Alonzo classification, includes three types of fractures^(^14^,^^15^^)^: type I (fracture of the upper part of the odontoid dens, above the level of the ligaments), which is usually considered stable; type II (the most common fracture, occurring at the base of the odontoid, below the cruciform ligament), which is frequently unstable; and type III (fracture involving the odontoid and the lateral masses), which is considered relatively stable.

### Statistical analysis

The frequency of upper cervical spine injuries, presence of intracranial injuries, and type of treatment were analyzed with Fisher's exact test. The relationship between head flexion-extension traumas and upper cervical spine injuries was analyzed with a one-sided binomial test. Values of *p* < 0.05 were considered statistically significant.

## RESULTS

**Figure 1:**
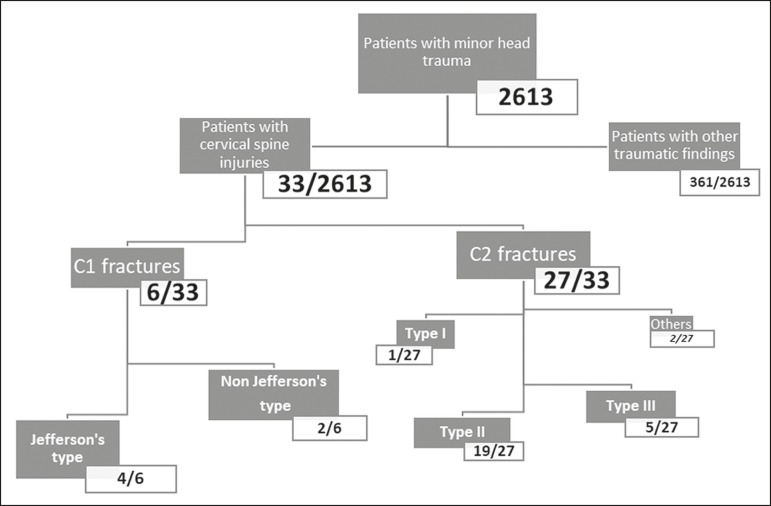
Baseline characteristics of the patients.

Of the 2613 patients evaluated, 1704 (65.21%) were women; 735 (28.13%) were 65-75 years of age; and 1878 (71.87%) were over 75 years of age. The mean age was 80.5 years in the sample as a whole, 70.7 years among the patients who were 65-75 years of age, and 84.3 years among the patients who were over 75 years of age. The most relevant baseline characteristics are summarized in Figure 1.

Among the 2613 patients, head CT showed no upper cervical spine injuries in 2065 (79.03%), relevant findings unrelated to trauma in 154 (5.89%), and relevant findings related to trauma in 394 (15.07%). Upper cervical spine injuries were identified in 33 (1.26%) of the sample as a whole, corresponding to 8.37% of the 394 patients with trauma-related findings.

We identified upper cervical spine injuries in six (0.82%) of the 735 patients who were 65-75 years of age and in 26 (1.38%) of those who were over 75 years of age. Fisher's exact test showed no significant differences between those two groups regarding the frequency of upper cervical spine injuries (*p* = 0.385) or the presence of associated intracranial injuries (*p* = 1), as well as in terms of the type of fracture or the treatment adopted (*p* = 0.058 for both).

Among the 33 patients with upper cervical spine injuries, associated intracranial injuries were observed in seven (21.2%): subarachnoid hemorrhage, in one patient; facial bone fractures, in four; and skull fractures, in two.

**Figure 2:**
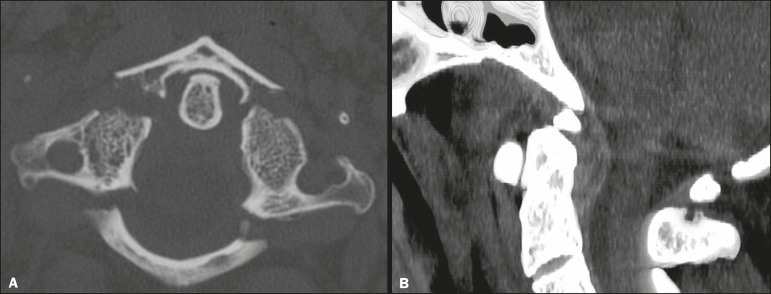
Head CT including the first two vertebrae in a 74-year-old patient with frontal trauma, a GCS score of 15, and no cervical symptoms. **A:** Axial image with a bone algorithm showing a Jefferson fracture of C1 involving the anterior and posterior vertebral arches. **B:** Sagittal reformatted image with a soft-tissue algorithm showing an accompanying anterior epidural hematoma.

**Figure 3:**
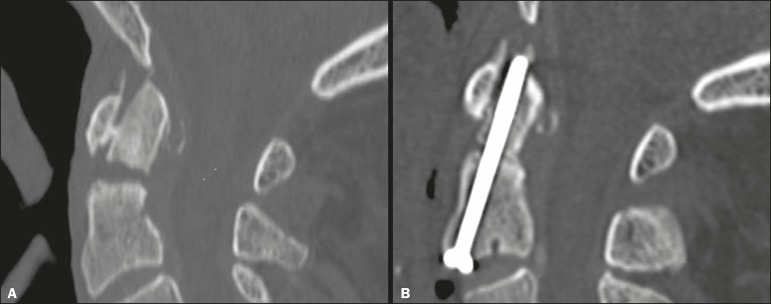
Head CT including the C1-C2 region in an 80-year-old patient with frontal trauma, a GCS score of 15, and no cervical symptoms. **A:** Sagittal reformatted image with a bone algorithm showing a type II odontoid fracture of C2 with diastasis of bone fragments and mild retroversion of the odontoid process. **B:** Postoperative volumetric CT showing successful treatment with anterior odontoid screw fixation.

**Figure 4:**
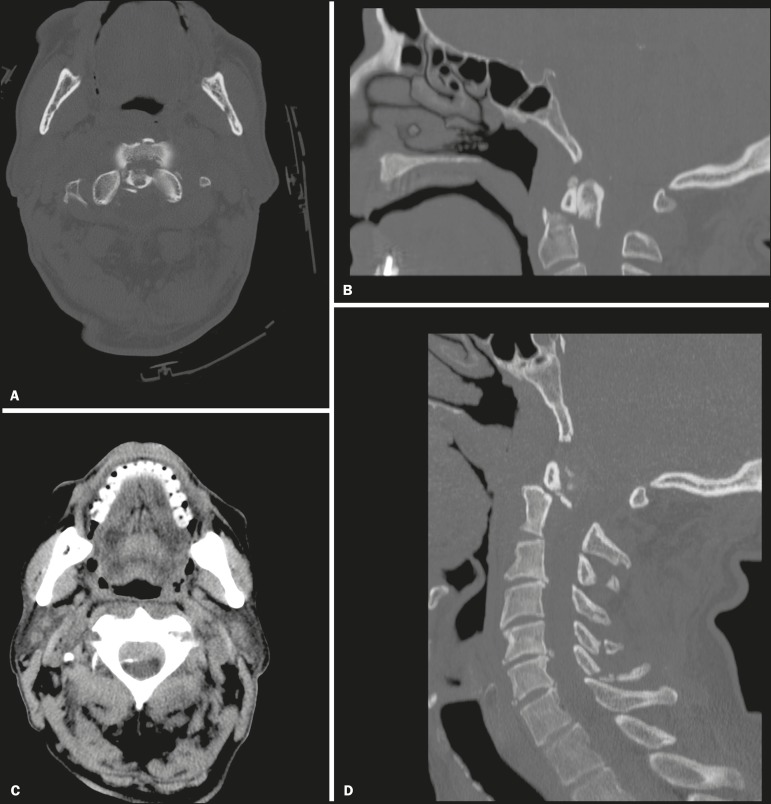
Head CT including the first two vertebrae in an 86-year-old patient with minor frontal head trauma, a GCS score of 15, and no cervical symptoms. **A,B:** Axial and sagittal reformatted images with a bone algorithm showing a type II odontoid fracture of C2 with diastasis of bone fragments and retroversion of the odontoid process. **C,D:** Associated epidural hematoma and fractures involving the spinous processes of the C4-C7 vertebrae (seen on a second CT scan of the entire cervical spine).

Six (18.2%) of the 33 patients had C1 fractures, and the remaining 27 (81.8%) had C2 fractures. Of the six patients with C1 fractures, four had a Jefferson fracture (Figure 2) and two had a non-Jefferson fracture. Of the 27 patients with C2 fractures, one had a type I odontoid fracture, 19 had a type II odontoid fracture, five had a type III odontoid fracture, and two had a fracture of the lateral mass or posterior arch. The most common upper cervical spine injury was type II odontoid fracture (Figures 3 and 4), which occurred in 19 (57.6%) of the 33 cases.

The therapeutic approach was conservative (immobilization with a cervical collar or halo vest) in 26 (78.8%) of the 33 patients. The remaining seven patients (21.2%) underwent surgical treatment: anterior odontoid screw fixation in three cases (Figure 3) and occipital-cervical fusion in four.

### Site and type of trauma in patients with upper cervical spine injuries

The site and type of trauma responsible for upper cervical spine injuries was unknown in two (6.1%) of the 33 affected patients. In the remaining 31 patients (93.9%), all of the upper cervical spine injuries were associated with frontal, occipital, or facial traumas. The one-sided binomial test revealed that frontal and flexion-extension head trauma were both significantly associated with upper cervical spine injuries (*p* < 0.001 for both).

### CT dose evaluation

Prior to the study outset, we used a CT head phantom, on both scanners, to calculate the CT dose and the volume-weighted CT dose index for our protocol (head and upper cervical spine) and for a standard head CT protocol. We assumed that a standard head CT scan would have a scan length of 15 cm, and that the inclusion of the first two cervical vertebrae would increase the total scan length to 19 cm, as measured on the CT scout of a male patient with a height of 1.74 cm. The effective dose (in mSv) was calculated with the ImPACT CT Patient Dosimetry Calculator, version 1.0.2 (ImPACT, London, England).

Because the volume-weighted CT dose index depends on the chosen exposure factors, scan field of view, collimation, and pitch factor, it was the same for both protocols. The effective dose for the standard head CT protocol was 5 mSv on the 16-slice scanner and 3.8 mSv on the 128-slice scanner. Inclusion of the first two cervical vertebrae increased the effective dose by 1.3 mSv (26.0%), making the total effective dose 6.3 mSv, and by 0.9 mSv (23.7%), making the total effective dose 4.7 mSv, on the 16-slice and 128-slice scanners, respectively.

## DISCUSSION

Traumatic brain injury encompasses a wide spectrum of injuries, especially in the elderly population. Most cases of trauma in the elderly are attributed to falls, and several studies have demonstrated that the incidence of falls in the United States increased by 120% in the last decade^(^16^)^. It is estimated that 30% of people ≥ 65 years of age fall from standing height each year, the majority with no serious damage. Although many injuries can lead to a poor outcome in elderly patients, recent studies suggest that timely, appropriate intervention leads to outcomes comparable to those seen in younger patients^(^16^)^.

There is no agreement about the use of CT in patients with minor head trauma, and several decision rules have been developed to predict the effective need for a CT scan, including economic considerations and the problem of radiation hazards^(^17^)^. In 2015, Easter et al. published a systematic review of the role of neuroimaging in adults with minor head trauma and a GCS score of 13-15. The authors proposed that, in patients at low risk, even in those who present one or more risk factor, clinical observation might be useful, and that such patients should undergo CT only if the signs or symptoms worsen^(^18^)^. There is more agreement about performing head CT in elderly patients with minor head trauma, given that they have a higher risk of intracranial hemorrhage and that no features of the personal history or physical examination can completely rule out intracranial injury^(^16^)^.

The incidence and frequency of cervical spine injuries are increasing as a consequence of the expansion of the geriatric population, related to the degenerative and osteoporotic effects that aging has on the upper cervical spine. The prompt, accurate diagnosis of cervical spine fractures might be difficult in the presence of extensive degenerative changes and deformities. In addition, elderly patients might sustain a cervical spine injury and not have specific neck pain. The absence of symptoms in elderly individuals, such as those in our sample, is perhaps due to the indirect mechanism of injury to the cervical spine or to an altered physiology and decreased mental status in traumatized elderly patients^(^19^-^21^)^. For all of these reasons, CT is considered the most cost-effective screening technique for cervical injuries, especially in the elderly. As previously mentioned, the NICE guidelines recommend that specific attention be given to the occipital condyle region in patients who have sustained a head injury^(^12^)^.

In the literature, there are few data regarding the incidence of upper cervical spine injuries in asymptomatic elderly patients with minor head trauma. By including the first two cervical vertebrae in our head CT scan protocol, we identified a relative high (1.26%) incidence of such injuries in elderly patients. Fractures of the C2 vertebra, predominantly type II odontoid fractures, constituted the most common injury. Among the 33 patients with upper cervical spine fractures, cranial injury was not common, occurring in only six cases, and spinal cord injury was extremely rare, occurring in only one. It is noteworthy that all of the patients with upper cervical spine injuries were asymptomatic, even in the presence of unstable fractures. We hypothesized that the high mean age of our patients could explain the lack of cervical symptoms. It is also important to highlight the fact that the geriatric population often presents several comorbidities and is subject to polypharmacy. Those conditions, together with cognitive impairment, which is common in that population, may lead to a paucity of reported symptoms. The consequence is that these upper cervical spine injuries would probably not have been diagnosed immediately, because X-ray and CT would not have been performed.

The discovery of upper cervical spine injuries is relevant for therapeutic decisions, because such injuries can cause cervical instability and can be accompanied by intraspinal hematomas or spinal cord injuries, with severe and potentially life-threatening sequelae. Most atlas fractures are stable and can be successfully managed by immobilization with a soft or hard collar. Unstable atlas fractures can also be treated conservatively by halo traction, although more and more surgeons now prefer surgery because of the potential discomfort and complications of halo traction^(^13^)^.

Treatment of odontoid fractures is controversial. In general, external immobilization (including traction, halo vest immobilization, and the use of a Philadelphia collar) is the preferred treatment for type I and III odontoid fractures. For type II odontoid fractures, conservative management has historically been associated with a high rate of nonunion in comparison with surgical intervention^(^22^-^24^)^.

Although nonsurgical treatment avoids the intrinsic risk of surgery, it is associated with a higher rate of nonunion, whereas surgical treatment improves the union rate, although there is no agreement in the literature regarding its effective survival advantage^(^23^)^. Considering specifically patients ≥ 65 years of age, previous studies have demonstrated a survival advantage of early surgery^(^22^)^. In the present study, surgical intervention was necessary in seven (21.2%) of the 33 patients diagnosed with cervical fractures. Most (57%) of the fractures were type II, which are considered unstable. However, in many cases, external immobilization with a collar was chosen over surgery because of the potential morbidity of surgical intervention, especially in older patients and in those with serious comorbidities.

It is also of note that upper cervical spine injuries were significantly associated with frontal, facial, and occipital head traumas. The flexion-extension movements of the craniocervical junction that occur during these types of traumas might be implicated in these type of injuries, even in those associated with low-energy trauma. It is known that hyperextension injury of the cervical spine is common in the elderly, typically affecting the mid and lower cervical levels^(^5^)^. However, to our knowledge, there have been no reports of an association between minor head trauma, craniocervical flexion-extension movements related to head trauma, and upper cervical spine injuries, which is relevant to our study.

Finally, considering the increase in CT dose, our protocol might be easily adopted because it differs from the standard head CT protocol by only a few centimeters in scan length and requires a dose increase of only 23-25%. In our opinion, that dose increase can be considered acceptable, given the age of these patients and the potential risks of a missed diagnosis.

In conclusion, elderly patients are at risk for minor head trauma and for cervical spine fractures, the majority being type II dens fractures. Our findings demonstrate that the inclusion of the first two cervical vertebrae in head CT scans performed in elderly individuals with minor head trauma helped identify a quite significant rate of upper cervical spine injuries, even in the absence of cervical symptoms. The frequency of cervical injury observed in our sample (1.26%) is relevant because these kinds of fractures have potentially life-threatening consequences. Our protocol changed the treatment plan for the affected patients with an acceptable increase in the effective dose.
